# Isolated Scalp Nodule in Patient with Undiagnosed RCC

**DOI:** 10.1100/tsw.2007.377

**Published:** 2006-05-17

**Authors:** David Pan, Owen Niall, Harbinder Sharma, Devan Gya

**Affiliations:** ^1^ Department of Urology, The Northern Hospital, Victoria, Australia; ^2^ General Surgery, The Northern Hospital, Victoria, Australia

**Keywords:** RCC, nodule, metastasis, cutaneous, nephrectomy

## Abstract

An unexpected diagnosis of metastatic RCC after excision biopsy of a skin nodule can bring uncertainty. A case of isolated scalp metastasis from undiagnosed RCC was noted and a review of the literature was undertaken to aid management. RCCs often present with distant disease involving multiple organ systems. Single metastasis to the scalp region without other organ involvement is uncommon. Cytoreductive nephrectomy and limited metastectomy offer survival advantage in physically fit patients with RCC.

## INTRODUCTION

Renal cell carcinoma (RCC) represents 3% of all human cancers and is the fourth most common cancer to metastasise. Most RCCs are now diagnosed incidentally on imaging, however, a minority can present with distant metastases. The prognosis for such a patient is poor. We report on a case of metastatic RCC presenting as a scalp nodule and review the relevant literature on management of advanced RCC.

## CASE

A 63-year-old, male, ex-smoker presented to the outpatient department for a 2-cm, itchy, scalp nodule. The nodule increased in size marginally over a 6-week period. There was no bleeding or discharge from the nodule. He had no urinary symptoms. A past history of cholelithiasis, peptic ulcer disease, dyslipidaemia, and TB were noted. All biochemistry was normal except marginally elevated serum creatinine. Excisional biopsy raised suspicion of metastatic RCC (Fig. 1).

Subsequent abdominal CT showed an 11-cm mass in his right kidney without evidence of extension into IVC or renal veins. CT scan of his chest and whole body bone scan were both unremarkable. The patient went on to have a radical nephrectomy and histopathology showed RCC of Fuhrman grade 3 (Fig. 2).

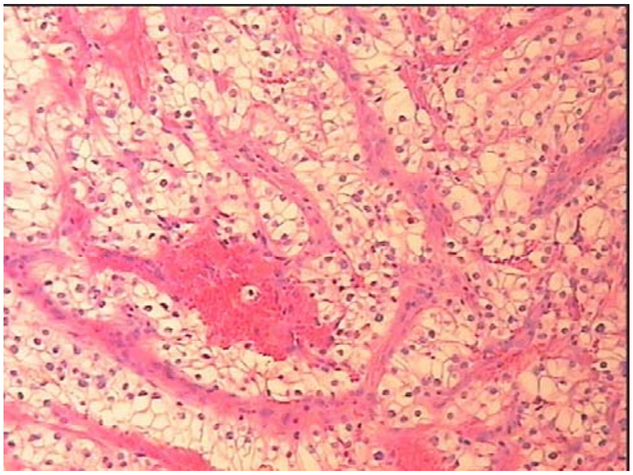

**FIGURE 1**. Microscopic examination of skin lesion at high power suggests clear cell carcinoma.

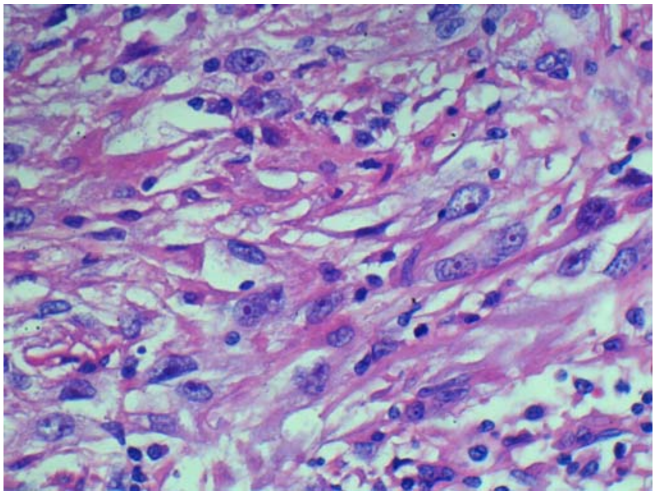

**FIGURE 2**. Microscopic examination at high power demonstrates Fuhrman Grade 3 RCC.

## DISCUSSION

Most RCCs are now detected incidentally with the increased use of imaging. The classic triad of RCC (haematuria, flank pain, mass) is seen in less than 10% of patients; 30% will present with skin, bone, and chest metastasis from undiagnosed RCC, and some of them have genitourinary symptoms[1]. RCC comprises 6.8% of all cutaneous metastasis from visceral cancers[2] and 82% of RCC metastasis involves multiple sites[5], most commonly lungs, bones, liver, abdominal skin, and central nervous system. The most common site for cutaneous metastasis from visceral cancers in general is the chest (28.4%) and scalp involvement occurs only in 7%[9]. Incidence of skin metastasis from RCC is 4%[9] and they ranked second in frequency of neoplastic skin metastasis just behind breast cancer (24%). Metastases to skin are solitary in 1.6–3.6% of patients[3] and 75% of patients with skin involvement had lung metastases at postmortem[4]. Incidence of metastasis increases considerably for RCCs with diameters of more than 5 cm.

There are numerous reports in the literature for cutaneous metastasis at variable intervals after nephrectomy, but undiagnosed RCC presenting as a solitary scalp lesion is rare. Kouroupakis[5] reported 3 patients who presented with solitary skin nodules, but 2 patients' symptomatology and other organ involvement were not documented. The remaining patient had lung metastasis as well. Mitsuhashi[6] noted 22 cases of RCC found after skin metastasis; 8 out of the 22 patients had multiple metastasis to the temple and scalp region, 3 out these 8 had other organ involvement at the time of cutaneous metastasis, and the remaining 5 patients' documentation was incomplete.

In 2001, the SWOG (Southwest Oncology Group) and EORTC (European Organisation for research and treatment of cancers) showed that cytoreductive nephrectomy increases survival in fit patients[7] and demonstrated a 5-month survival advantage in patients who received both nephrectomy and immunotherapy compared to immunotherapy alone. A study by Kavolius[8] shows a 5-year survival of 44% after combining metastectomy and nephrectomy, which compares favourably with the average survival of 4–20%. For unfit patients, however, systemic immunotherapy remains to be the primary mode of therapy. The value of consolidative surgery in those patients who respond to immunotherapy is still questionable.

## CONCLUSION

RCCs presenting as isolated scalp metastasis is rare. The clinician needs to have a high index suspicion because of the high metastatic potential of RCCs. Fit patients with limited metastatic disease should have the option to receive cytoreductive nephrectomy and metastectomy with or without immunotherapy to extend their survival. Unfit patients will continue to have immunotherapy as the primary mode of therapy.

## ACKNOWLEDGEMENTS

Special thanks to Dr. Sam Kolber, FRACP(Path), who provided histology of the skin biopsy in digital form.
